# Broadband Acoustic Sensing with Optical Nanofiber Couplers Working at the Dispersion Turning Point

**DOI:** 10.3390/s22134940

**Published:** 2022-06-30

**Authors:** Xu Gao, Jiajie Wen, Jiajia Wang, Kaiwei Li

**Affiliations:** 1 College of Instrumentation & Electrical Engineering, Jilin University, Changchun 130000, China; gaox19870513@163.com; 2 School of Opto-Electronic Engineering, Changchun University of Science and Technology, Changchun 130022, China; wenjiajie9504@163.com; 3 College of Agricultural Equipment Engineering, Henan University of Science and Technology, Luoyang 471003, China; jjw@haust.edu.cn; 4 Key Laboratory of Bionic Engineering of Ministry of Education, Jilin University, Changchun 130022, China

**Keywords:** optical fiber coupler, acoustic sensing, dispersion turning point

## Abstract

Herein, a broadband ultrasensitive acoustic sensor based on an optical nanofiber coupler (ONC) attached to a diaphragm is designed and experimentally demonstrated. The ONC is sensitive to axial strain and works as the core transducing element to monitor the deformation of the diaphragm driven by acoustic waves. We first theoretically studied the sensing property of the ONC to axial strain and the deformation of the diaphragm. The results reveal that ONC working at the dispersion turning point (DTP) shows improved ultra-sensitivity towards axial strain, and the largest deformation of the circular diaphragm occurs at the center. Guided by the theoretical results, we fabricated an ONC with a DPT at 1550 nm, and we fixed one end of the ONC to the center of the diaphragm and the other end to the edge to construct the acoustic sensor. Finally, the experimental results show that the sensor can achieve accurate measurement in the broadband acoustic wave range of 30~20,000 Hz with good linearity. Specifically, when the input acoustic wave frequency is 120 Hz, the sensitivity reaches 1923 mV/Pa, the signal-to-noise ratio is 42.45 dB, and the minimum detectable sound pressure is 330 μPa/Hz^1/2^. The sensor has the merits of simple structure, low cost, and high performance, and it provides a new method for acoustic wave detection.

## 1. Introduction

Acoustic wave sensors play a dominant role in medical diagnosis, earthquake prediction, and volcano monitoring. Compared with the traditional magnetoelectric acoustic sensors, optical fiber acoustic sensors have the advantages of small size, light weight, high sensitivity, and strong anti-electromagnetic interference ability [1]. In recent years, researchers have proposed many acoustic sensing technologies based on the principle of optical fiber sensing. Depending on differences in the sensor configuration and mechanism of acoustic-to-optical signal conversion, these sensors can be divided into two categories. One is the fiber-optic Fabry–Pérot cavity, which is constructed of a fiber tip and a reflective diaphragm that can convert acoustic waves to changes in optical cavity length and, hence, the shift in the optical interference signal via its mechanical deformation [2,3,4,5,6,7]. The other type is based on a strain sensor that is attached to a diaphragm/plate and is responsible for converting the acoustic vibration to the axial elongation of the fiber optic strain sensor and, hence, the optical signal [8,9,10,11].

In the past decades, a variety of diaphragm materials, such as silicon [2], polymer [3], graphene [4,5], chitosan [6], and silver [7], have been studied for fiber-optic Fabry–Pérot cavity acoustic sensors. In addition, reducing the thickness-to-diameter ratio of the diaphragm can significantly improve the sensitivity of such acoustic sensors [1]. However, the production processes of polymer film, metal film, and micromachined silicon film are complex and expensive, involving mechanical spinning, chemical etching, or micromachining. Further, the requirements for demodulation are relatively high and costly.

The second type of fiber optic acoustic sensor has also undergone rapid development in recent years. Sumit Dass et al. designed an acoustic sensor based on a single-mode tapered fiber and a butyronitrile thin diaphragm, achieving a minimum detectable sound pressure of 21.11 Pa/Hz^1/2^ and flat output response at frequencies of 250 Hz~2500 Hz [8]. Shun Wang et al. proposed an optical fiber acoustic sensor based on a nonstandard fused coupler and aluminum foil, which achieved a sensitivity of up to 2.63 mW/Pa and an acoustic measurement range of 20 Hz~20 kHz [9]. Wenjun Ni et al. proposed a thin-core ultra-long-period fiber-grating-based acoustic wave sensor showing an operation range of 1 Hz–3 kHz and high sensitivity up to 1890 mv/Pa at 5 Hz [10]. However, some of the above-mentioned acoustic wave sensors cannot achieve wide-band acoustic wave measurement. Also, the sensitivities are usually relatively low, and preamplifiers are required to achieve high sensitivity. Therefore, exploiting new optical fiber acoustic sensors with broad working frequency ranges and high sensitivity still attracts the wide attention of researchers. Recently, Kaijun Liu et al. demonstrated that the microfiber Mach–Zehnder interferometer working at the dispersion turning point (DTP) can be employed for vibration detection, and ultra-high sensitivity was achieved [11].

We recently discovered and demonstrated the dispersion turning point (DTP) in optical nanofiber couplers (ONCs), which can improve the sensing performance [12,13]. ONCs working at the DTP show enhancements of nearly two orders of magnitude compared with conventional fiber coupler sensors for refractive index (RI) sensing [12,14], temperature sensing [13], biochemical sensing [15], and axial strain sensing [16]. This study further implements the ONC with a DTP in acoustic sensing. The acoustic sensor was constructed by fixing the fiber coupler on a specially designed sensing diaphragm. Acoustic waves drive the sensing diaphragm to vibrate, and the vibration changes the coupling length of the ONC dynamically and is measured by the ONC, which works as an ultra-sensitive tensile sensor. We first theoretically calculated and analyzed the strain sensing performance of the ONC operation at the DTP. Then, we numerically studied the acoustic induced vibration characteristics of the polyethylene (PE) diaphragm. Finally, we experimentally realized a sensing device that can achieve acoustic wave measurement of 30–20 kHz and achieved a high sensitivity of 1929 mV/Pa at 120 Hz. The sensor has a simple structure, low requirements for demodulation equipment, and low cost, which provides a new method for acoustic wave detection.

## 2. Principle of Acoustic Sensing Using the ONC–Diaphragm Configuration

**Figure 1:**
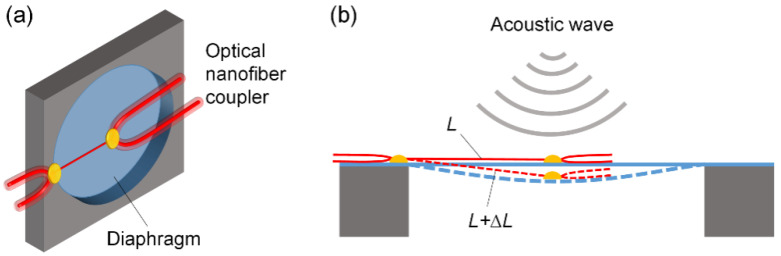
(**a**) Structure diagram of the optical-nanofiber-based acoustic sensor. (**b**) The acoustic wave sensing principle of the diaphragm-supported optical nanofiber sensor.

The configuration of the proposed ONC-based acoustic sensor is shown in Figure 1a. An ONC that operates at the DTP was adopted as the core sensing element, with one end fixed at the center of a PE diaphragm and the other end fixed on the edge of the diaphragm. As shown in Figure 1b, the dynamic acoustic wave drives the diaphragm to vibrate accordingly, and the vibration imposes stress on the ONC dynamically, leading to variation in the coupling length *L*. As the ONC sensor working at the DTP is quite sensitive to changes in length, the acoustic wave of slight sounds can be readily detected by tracing the optical output signal of the ONC sensor.

### 2.1. Working Principle of the ONC

**Figure 2:**
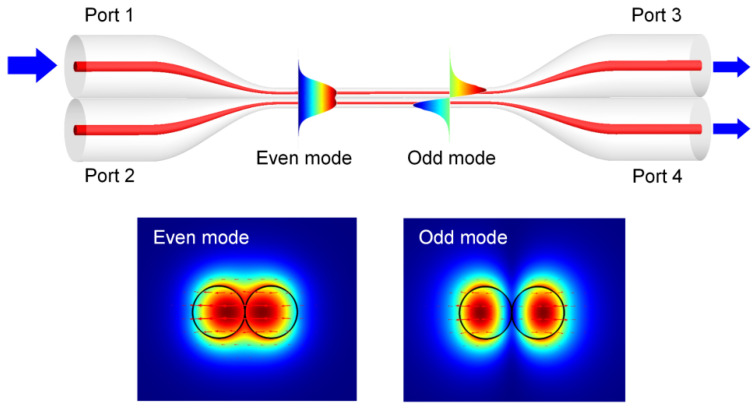
Schematic diagram of the nanofiber coupler and the modal field distributions for the even mode and odd mode.

As the sensing of acoustic waves is realized by stretching the fiber coupler, we first analyze the strain sensing performance of the ONC numerically. The configuration of the ONC is shown in Figure 2. The ONC is formed by fusion splicing two single-mode fibers, including a coupling section, two input ports, and two output ports. The coupling section of the two microfibers forms a new waveguide. When light enters the conical transition region from Port 1, both the even mode and odd mode can be excited simultaneously. The two modes propagate along the waveguide and gradually accumulate phase difference, finally coupling to the two output ends to produce mode interference. When the coupling section is stretched, the length of the optical path varies and the interference fringes of the output spectrum shift.

Assume that the input optical power at Port 1 is *P*_0_ at a certain wavelength $\lambda_{N}$. Thus, the output energy of output Ports 3 and 4 can be obtained as [13]:

(1) $$P_{3}=P_{0}\cos^{2}\left(\frac{1}{2}\phi \right),$$

(2) $$P_{4}=P_{0}\sin^{2}\left(\frac{1}{2}\phi \right)$$

The phase difference *ϕ* between the two modes satisfies:

(3) $$\phi_{N}=\frac{2\pi L\left(n_{\mathrm{eff}}^{\mathrm{even}}-n_{\mathrm{eff}}^{\mathrm{odd}}\right)}{\lambda_{N}}=\left(2N-1\right)\pi$$

where $n_{\mathrm{eff}}^{even}$ and $n_{\mathrm{eff}}^{odd}$ are the effective refractive index values (ERIs) of the odd mode and even mode. *L* is the length of the uniform coupling section, and *N* is an integer. In this study, we only consider the uniform waist region as it is much thinner and much longer than the tapered region, and the waist region is much more sensitive than the tapered region.

When external stretching is applied to the nanofiber coupler, the waist segment undergoes a prolongation along the axial direction and shrinkage in the radial direction. Concurrently, the RI of the waist region also undergoes a decrease as a result of the elasto-optic effect. The shrinkage in diameter and decrease in RI change the ERIs of the two guided modes, and the prolongation of the waist modifies the coupling length. As a result, the phase difference *ϕ* experiences a variation and finally reflects the change in the output light intensity of *P*_3_ and *P_4_*. The tensile strain of the optical fiber is *δ = ∂L/L*. Thus, the sensitivity of the corresponding coupler strain can be deduced as [16]:

(4) $$S_{\delta }=\frac{\partial \lambda_{N}}{\partial \delta }=\frac{\lambda_{N}}{n_{g}^{\mathrm{even}}-n_{g}^{\mathrm{odd}}}\left(\Delta n_{\mathrm{eff}}+\frac{\partial \Delta n_{\mathrm{eff}}}{\partial \delta }\right)$$

where $\Delta n_{\mathrm{eff}}=n_{\mathrm{eff}}^{\mathrm{even}}-n_{\mathrm{eff}}^{\mathrm{odd}}$, $n_{g}=n_{eff}-\lambda_{N}\partial n_{eff}/\partial \lambda$ represents the group ERI of the guided mode, and $G=n_{g}^{\mathrm{even}}-n_{g}^{\mathrm{odd}}$ denotes the group ERI difference between the two modes. It should be noted that the term $\partial \left(\Delta n_{\mathrm{eff}}\right)/\partial \delta )$ is dependent on both the elasto-optic coefficient and the Poisson’s ratio of fused silica. Equation (4) reveals that when G=0, the sensitivity of the nanofiber coupler to axial strain can be improved significantly. The wavelength which satisfies G=0 is also known as the dispersion turning point, and it can only be satisfied when the group ERIs of the two modes equal each other.

**Figure 3:**
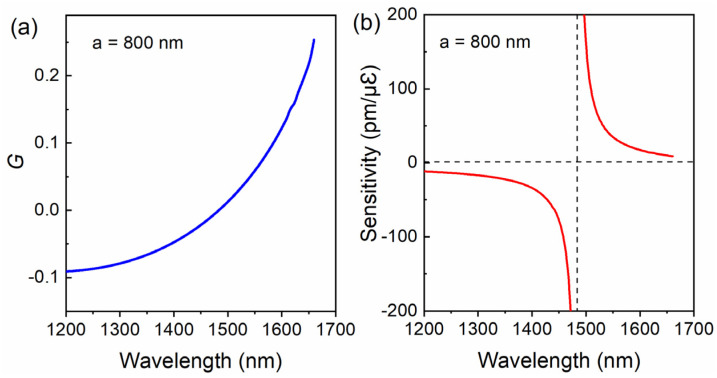
(**a**) Effective refractive index difference between the odd and even modes. (**b**) The strain sensitivity of the ONC.

In order to gain a straightforward understanding of the sensing performance for the optical nanofiber coupler to axial strain and, hence, acoustic waves, we carried out numerical simulations. We first calculated the group ERI difference *G* between the odd mode and the even mode for a nanofiber coupler with a diameter of 1.6 μm, within the wavelength range of 1200~1700 nm. As shown in Figure 3a, the group ERI difference *G* varies from a negative value to a positive value as the working wavelength gradually increases from 1200 nm to 1660 nm, and it is equal to 0 at a wavelength of about 1482 nm. This critical wavelength is the DTP for the nanofiber coupler with a diameter of 1.6 μm. According to Equation (4), the axial strain sensitivity can reach infinity when *λN* is divided by 0.

Then, we calculated the axial strain sensitivity of the sensor according to Equation (4). The calculation results in Figure 3b reveal that the sensitivity curve shows a rectangular hyperbola shape. The axial strain sensitivity is significantly enhanced towards −∞ on the left side of the DTP and towards +∞ on the right side. This means that when interference dips/peaks approach the DTP, the wavelength shifts induced by axial strain can be greatly enhanced. According to our previous research, the DTP for nanofiber couplers in air can be tuned from 940 to 1670 nm simply by increasing the fiber diameter from 500 to 900 nm. By utilizing the DTP, ultrahigh sensitivity of nearly 100 nm/με can be achieved, which is promising for acoustic wave sensing and vibration measurement applications.

### 2.2. Working Principle of the Diaphragm

Due to the small diameter of the ONC, it is difficult for sound pressure to act on the ONC itself. So, a diaphragm is used as the sound-pressure-sensitive element to convert the acoustic wave to the mechanical vibration of itself. The ONC attached to the diaphragm can sense the acoustic wave indirectly by monitoring the vibration of the diaphragm. When the acoustic wave acts on the sensing diaphragm, the diaphragm will deform. When the pressure applied to the diaphragm is FP, the bending deformation of the diaphragm is [17,18]:

(5) $$d=\frac{3F_{P}R^{4}\left(1-\mu^{2}\right)}{64Eh^{3}}{\left(1-\frac{r^{2}}{R^{2}}\right)}^{2}$$

where *μ* and *E* are the Poisson’s ratio and Young’s modulus of the diaphragm material, respectively. *R* and *h* denote the radius and thickness of the diaphragm, respectively, and *r* denotes the distance from a point on the diaphragm to the center of the diaphragm.

**Figure 4:**
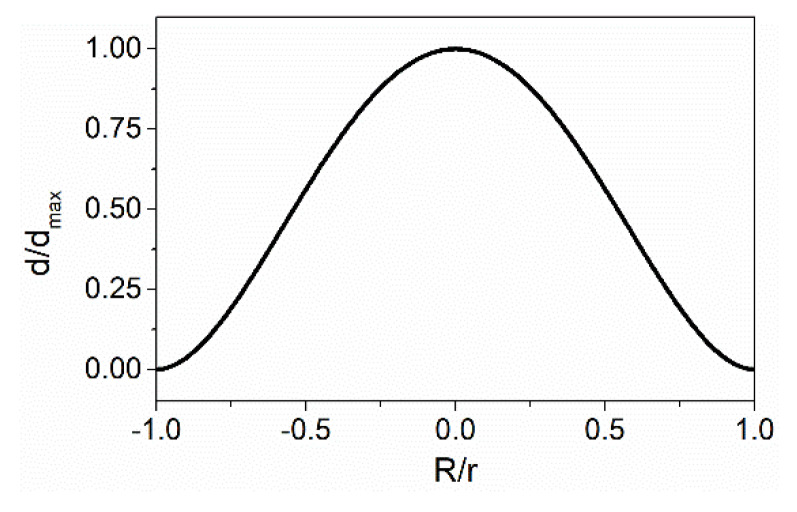
Normalized deformation at any point of the diaphragm, as a function of the normalized distance to the center.

According to (5), the cross-sectional curve of the deformation for a circular diaphragm can be obtained, as shown in Figure 4. It can be seen that the maximum deformation occurs at the center of the diaphragm. This indicates that to obtain a higher acoustic sensitivity, one end of the ONC should be fixed at the center of the diaphragm, and the other end should be fixed at the edge. In this way, the optical fiber coupler has the maximum deformation when the acoustic wave acts on the diaphragm.

Then, we analyzed the broadband acoustic response characteristics of the diaphragm. The selected diaphragm is a circular PE diaphragm with a radius of 4 cm and a thickness of 50 μm. The resonance frequency of the diaphragm is [17,18]:

(6) $$f_{mn}=\frac{k_{mn}^{2}h}{4\pi hR^{2}}\sqrt{\frac{E}{3\rho \left(1-\mu^{2}\right)}}$$

where the *k_mn_* value of the one-dimensional circular diaphragm is 3.196; *R* and *h* are the radius and thickness of the diaphragm, respectively. *E* and *ρ* are the Young’s modulus and density of the diaphragm, respectively.

**Figure 5:**
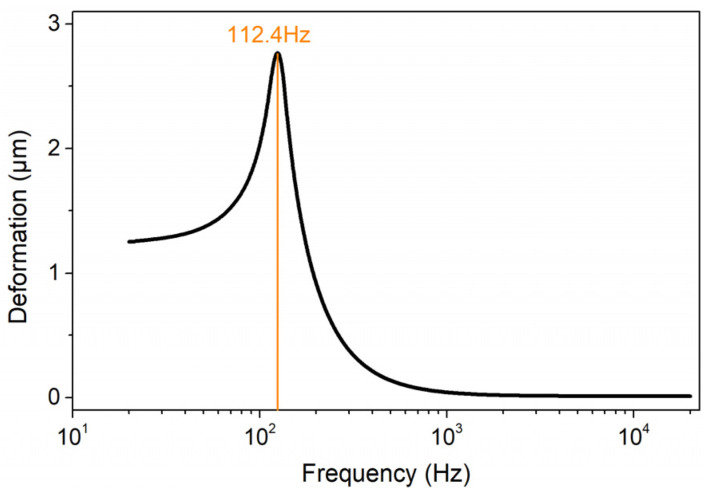
Diaphragm deformation diagram under different frequency acoustic waves when the pressure is 1 Pa.

The dynamic deformation of the diaphragm is [17,18]:

(7) $$\Delta d=\frac{3\left(1-\mu^{2}\right)R^{4}P}{16Eh^{3}}\frac{f_{mn}^{2}}{\sqrt{{\left(f_{mn}^{2}-f_{a}^{2}\right)}^{2}+4f_{a}^{2}\beta^{2}}}$$

where *β* is the damping coefficient, $f_{a}$ is the acoustic wave frequency, and *P* is the sound pressure. The calculation results are shown in Figure 5, which indicates that the diaphragm has the largest deformation when the acoustic wave is about 122.4 Hz, and the deformation is relatively gentle at 500 Hz–20 kHz.

## 3. Sensor Fabrication and Acoustic Measurement System

**Figure 6:**
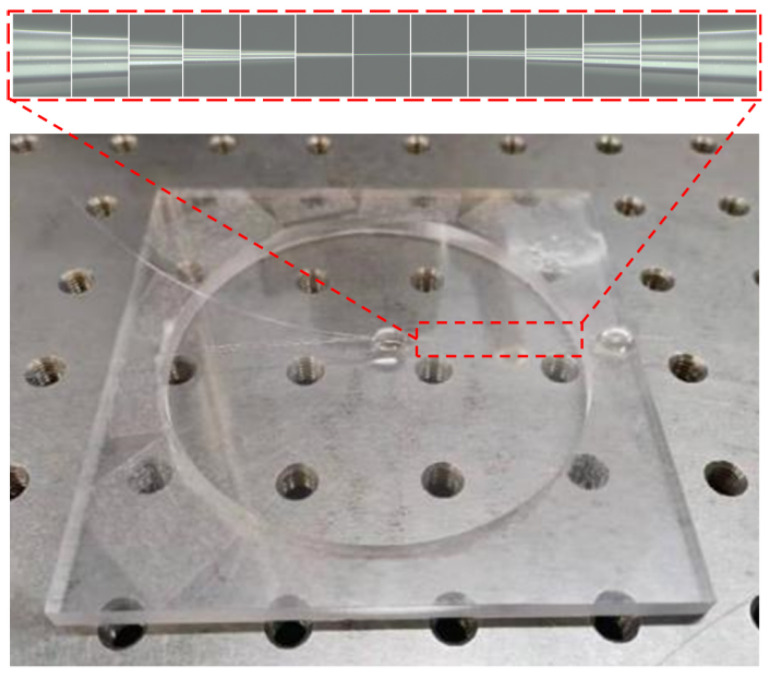
Photograph of the nanofiber coupler acoustic sensor.

Optical nanofiber couplers were fabricated from standard telecommunication single-mode optical fibers by the fusion elongation method. Generally speaking, two sections of bare single-mode fibers were double twisted and fixed by two fiber clamps. Then, the fibers were heated to the glass transition temperature by an alcohol lamp, and two motorized translation stages stretched the fibers. The two optical fibers shrank in diameter and were fused together; finally, the nanofiber coupler was obtained. To ensure that the optical nanofiber couplers possessed a DTP at the desired wavelength, an online monitoring system was used to track the output spectrum of the fiber coupler in real time. The tapering process was terminated once the DTP appeared at the desired wavelength in the output spectrum. Figure 6 depicts a representative micrograph of a fabricated optical nanofiber coupler.

The pedestal for the PE diaphragm was fabricated by a CNC machining center. The radius for the central hole was 4 cm. The PE film was fixed to the pedestal using UV glue, and a circular diaphragm with a radius of 4 cm was formed. Then, we fixed one end of the fabricated nanofiber coupler at around the center of the diaphragm and the other end at the edge with UV glue. A photograph of the fabricated acoustic wave sensor is shown in Figure 6.

**Figure 7:**
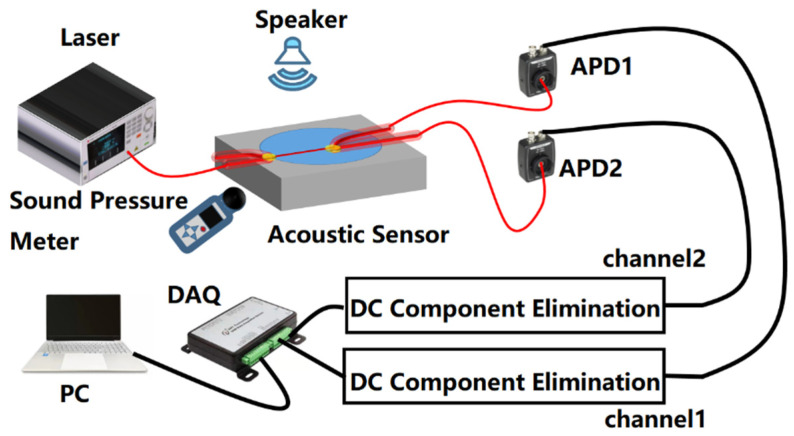
Schematic diagram of the acoustic wave measurement system.

The acoustic wave measurement system for the optical nanofiber coupler sensor is shown in Figure 7. The system consists of a sensing module and a signal demodulation module. The sensing part includes a tunable band laser (SANTEC TSL-550, Santec Corporation, Komaki, Aichi, Japan), the nanofiber coupler acoustic sensor, and two avalanche photodiodes (APDs, THORLABS PDA20C2, Thorlabs, Newton, NJ, USA). The signal demodulation part includes the filtering and amplifying circuits, a data acquisition card (DAQ, ART usb3133A, ART Technology, Beijing, China), and a computer. The light emitted by the laser enters one input arm of the nanofiber coupled acoustic wave sensor, and the optical signals from the two output arms are detected by the APDs. Then, after filtering, the data are collected by the DAQ, and finally, the signal is displayed, analyzed, and processed by the computer. Acoustic wave signals with the desired waveform and frequency were generated by a speaker driven by a function generator. A commercial sound pressure meter (SMART SENSOR AS824, Walfront LLC., Lewes, DE, USA) was used to measure the sound pressure of the acoustic wave signal.

## 4. Experimental Results and Discussion

**Figure 8:**
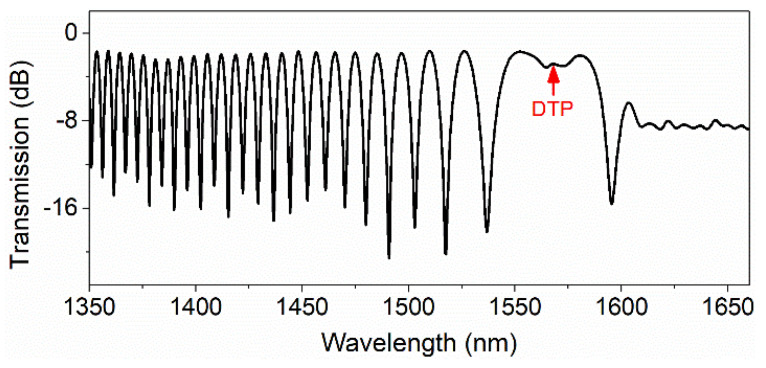
Spectrogram of the fabricated ONC with the DTP.

**Figure 9:**
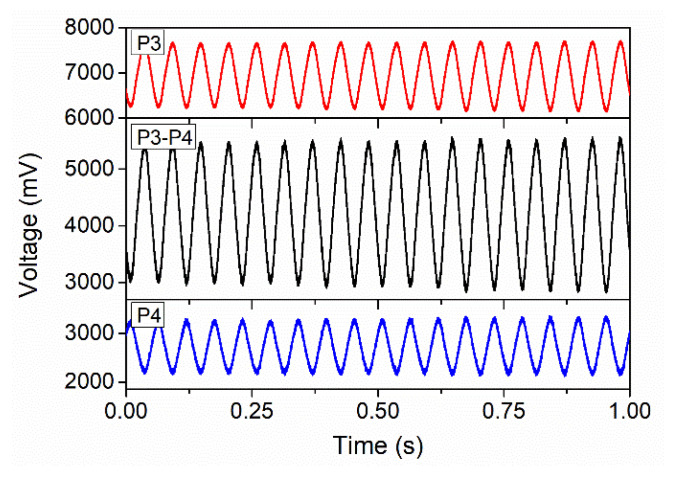
Measured signals from Ports 3 and 4 and the difference between Ports 3 and 4 under a 40 Hz acoustic wave.

The output spectrum from Port 3 of the ONC is displayed in Figure 8. It can be seen that the light wavelength corresponding to the DTP is about 1573 nm. Therefore, the output wavelength of the laser was set to 1573 nm, and this wavelength was chosen as the working wavelength of the ONC-based acoustic sensor. To improve the sensitivity for acoustic wave measurement, the difference between the output signals from Port 3 and Port 4 was used to measure acoustic waves. Figure 9 displays the detected signals from Ports 3 and 4 and their difference for acoustic signals of 40 Hz. It can be seen from the figure that the signal amplitude increased significantly after the difference operation.

### 4.1. Sensing Performance for Different Sound Pressures

**Figure 10:**
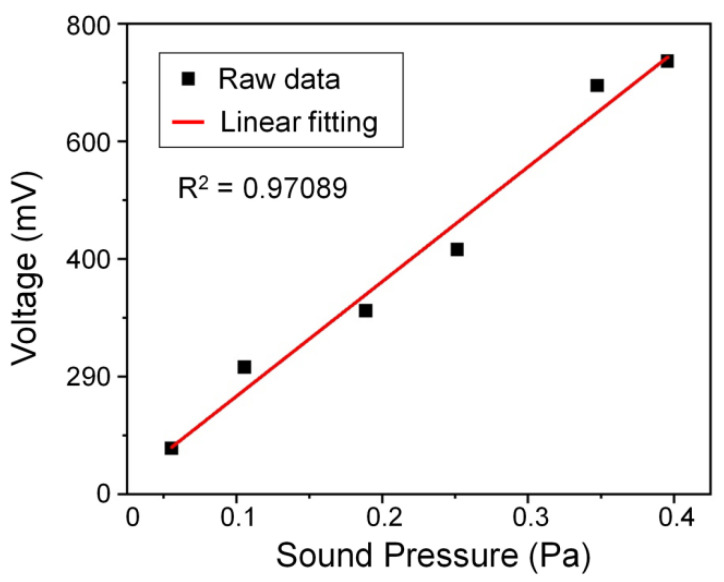
Linear diagram of output voltage under different sound pressures.

First, we evaluated the performance of the nanofiber coupler acoustic sensor in response to different sound pressures. We applied an acoustic wave with a frequency of 160 Hz to the sensor and varied the loudness of the acoustic wave sequentially. The sound pressure was measured using a sound pressure meter. The output signal from the acoustic sensor is summarized in Figure 10, proving that the output voltage of the sensor acoustic signal is linear with the input sound pressure.

### 4.2. Broadband Acoustic Detection

Then, we analyzed the broadband acoustic detection capability of the nanofiber coupler acoustic sensor. We loaded acoustic wave signals with frequencies ranging from 30 Hz to 20 kHz on the sensor diaphragm and obtained the time-domain output signal using the demodulation system. We performed an FFT transformation on the time-domain signals to obtain the frequency-domain diagram of the different signals.

**Figure 11:**
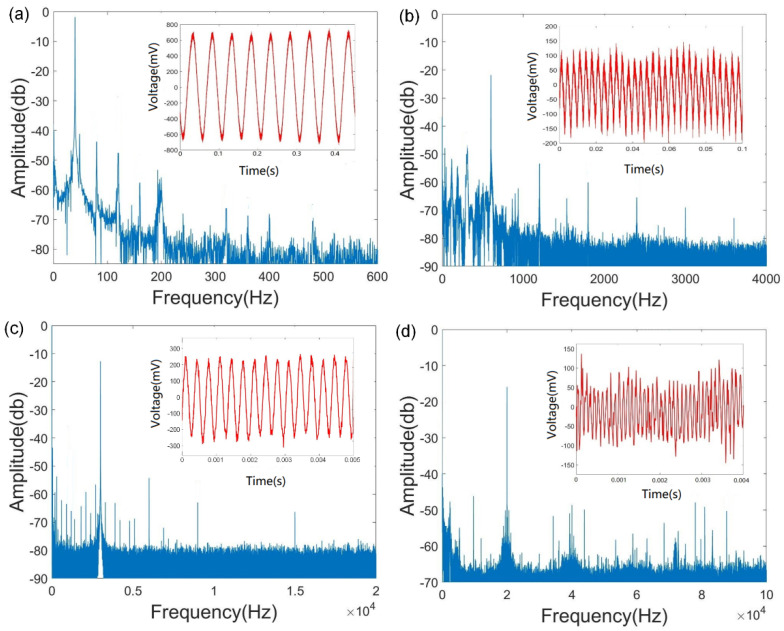
(**a**–**d**) Time-domain and frequency-domain diagrams of the sensor at frequencies of 30 Hz, 600 Hz, 3000 Hz, and 20,000 Hz, respectively.

The proposed optical-nanofiber-coupler-based acoustic wave sensor shows a good response to acoustic waves in the broad frequency range of 30 Hz–20 kHz. The representative measured time-domain signals and frequency-domain diagrams for acoustic waves with frequencies of 30 Hz, 600 Hz, 3000 Hz, and 20,000 Hz are shown in Figure 11. The results reveal that our sensor shows a high signal-to-noise ratio in this wide frequency range.

The sensitivity of our sensor in this broadband frequency range was calculated based on the experimental results. As a comparison, we also tested the performance of our sensor with working wavelengths of 1500 nm and 1600 nm, respectively. One of these two working wavelengths lies on the left side of the DTP, and the other lies on the right side. According to the results of our numerical investigation, the optical nanofiber coupler sensor would show inferior performance working at these two wavelengths as compared to working at the DTP. The sensing performance in the frequency range of 30 Hz–20 kHz was measured using the demodulation system, and the sensitivities were also calculated.

**Figure 12:**
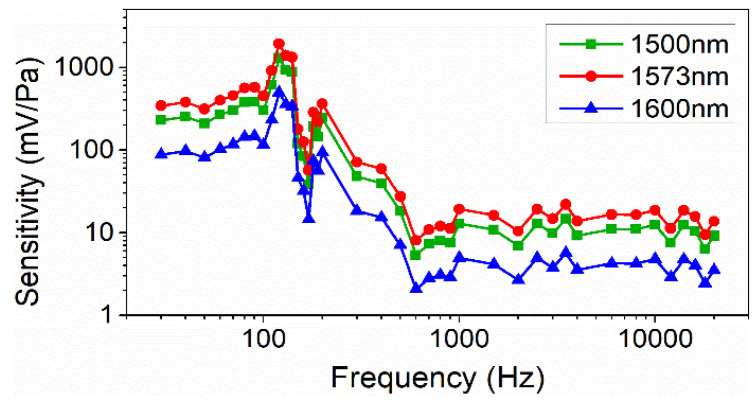
Sensitivity curves at different acoustic frequencies.

All the results are displayed in Figure 12. After comparing and analyzing the respective sensitivities of the sensor working at 1500 nm, 1573 nm, and 1600 nm, it was found that the sensor showed higher sensitivities in the measured frequency range when working at the DTP than when working at the other two wavelengths. These results are consistent with our numerical results. Also, the sensor showed relatively high sensitivities in the low-frequency range of 30~500 Hz. The highest sensitivity was discovered at the frequency of 120 Hz, which agrees well with our numerical simulation result of 112.4 Hz. The small discrepancy may be induced by the UV glue and the optical nanofiber coupler that is fixed at the center of the diaphragm. The sensitivity change was relatively stable from 500 Hz to 20 kHz. Currently, the sensitivity is between 8 and 30 mv/Pa without the amplifier circuit.

**Figure 13:**
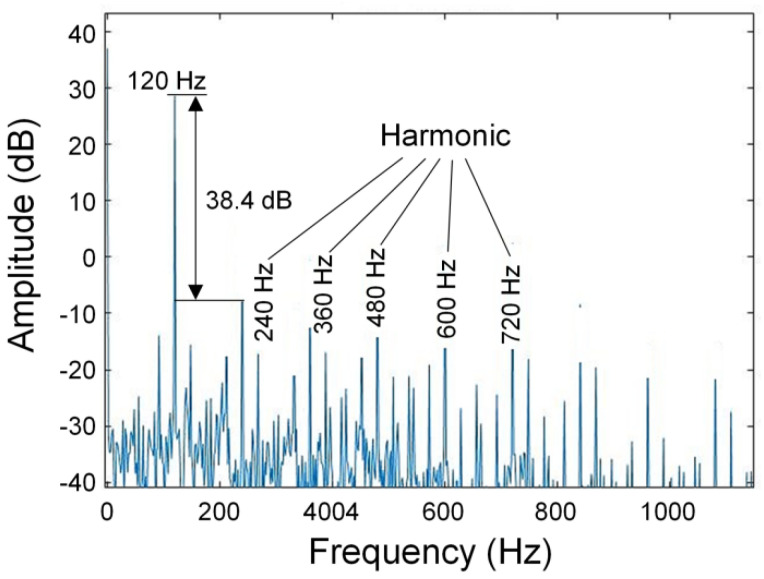
Frequency-domain diagram of the measured acoustic signal (operation wavelength: 1573 nm, acoustic wave frequency: 120 Hz).

We further analyzed the sensing performance of the sensor when a 120 Hz acoustic wave signal was loaded, as the sensor showed the highest sensitivity at this frequency. The frequency spectrum obtained through FFT transformation is shown in Figure 13. The prominent peak at 120 Hz corresponds to the frequency of the applied acoustic signal. The signal-to-noise ratio of the sensor is 38.4 dB, the minimum detectable sound pressure is 330 μPa/Hz^1/2^, and the sensitivity is 1923 mV/Pa. Harmonic signals also appeared in the frequency spectrum, which may be caused by the reflection of acoustic waves in the cavity of the sensor base between the diaphragm and the test bench. These harmonic signals are negligible compared to the fundamental frequency signal. Our study demonstrates that optical nanofiber couplers working at the DTPs are promising candidates for developing high-sensitivity acoustic wave sensors.

## 5. Conclusions

In summary, in this paper, we proposed an ultrasensitive acoustic wave sensor based on a micro/nanofiber coupler operating at the dispersion turning point and a sensing diaphragm. The sensor was realized by fixing the fiber coupler on the diaphragm and converting the vibration of the diaphragm to the stretching of the fiber coupler. Our theoretical studies showed that the sensing performance can be improved significantly by utilizing the DTP, and the diaphragm can provide a broadband response to acoustic waves. Guided by our theoretical findings, we experimentally realized ultra-high sensitivity and broadband detection of acoustic waves using a nanofiber coupler working at the DTP and a sensing diaphragm. The sensor shows high sensitivity in low-frequency measurement and flat response to medium- and high-frequency acoustic signals, with the highest sensitivity of 1929 mV/Pa achieved at 120 Hz. This sensor has the advantages of being simple in construction and easy for demodulation, and it may have potential applications in seismic wave detection.

## Data Availability

The datasets are available from the corresponding author on reasonable request.
